# FLUORESCENCE LIFETIME IMAGING OPHTHALMOSCOPY: Findings After Surgical Reattachment of Macula-Off Rhegmatogenous Retinal Detachment

**DOI:** 10.1097/IAE.0000000000002718

**Published:** 2019-12-19

**Authors:** Damian Jaggi, Yasmin Solberg, Chantal Dysli, Andreas Ebneter, Sebastian Wolf, Martin S. Zinkernagel

**Affiliations:** * Department of Ophthalmology, Inselspital, Bern University Hospital, Bern, Switzerland; and; † Department of BioMedical Research, University of Bern, Bern, Switzerland.

**Keywords:** fluorescence lifetime imaging ophthalmoscopy, FLIO, retinal detachment, macula-off, ophthalmic imaging

## Abstract

**Purpose:**

The purpose of this study was to investigate fluorescence lifetime imaging ophthalmoscopy lifetimes after macula-off rhegmatogenous retinal detachment (RRD) repair.

**Methods:**

Fifty-eight patients with successful macula-off RRD reattachment surgery were included. Retinal autofluorescence was excited with 470 nm, and amplitude-weighted mean fluorescence lifetimes (Tm) were measured in a short spectral channel (SSC, 498–560 nm) and a long spectral channel (LSC, 560–720 nm). Tm were obtained within a standardized Early Treatment Diabetic Retinopathy Study grid and correlated with Tm. The unaffected fellow eye served as control.

**Results:**

Fifty-eight patients (age: 65 ± 1.6 years, 11 women) were imaged at median 1.5 months postoperatively. Tm were significantly prolongxxxed within areas of previously detached retina in the long spectral channel and particularly in the central subfield in the short spectral channel. Short lifetimes in the center of the Early Treatment Diabetic Retinopathy Study grid correlated with better visual acuity (short spectral channel; r^2^ = 0.18, *P* = 0.001, long spectral channel; r^2^ = 0.08, *P* = 0.03). Areas of residual subretinal fluid pockets in four RRD eyes displayed short fluorescence lifetimes.

**Conclusion:**

Areas of previously detached retina exhibit significant fluorescence lifetime changes. We found a significant correlation of fluorescence lifetimes within the fovea with visual acuity after successful RRD repair. Our data suggests that the prolongation of fluorescence lifetimes in the fovea is mainly driven by loss of macular pigment. Therefore, fluorescence lifetime imaging ophthalmoscopy may be useful in the prediction of long-term functional outcomes after macula-off RRD surgery.

## Abstract 2

This study confirms that fluorescence lifetime imaging ophthalmoscopy is able to identify and quantify macular alterations after surgical reattachment of macula-off rhegmatogenous retinal detachment that relate to visual acuity. Fluorescence lifetime imaging ophthalmoscopy could be a useful noninvasive diagnostic tool to assess eyes after rhegmatogenous retinal detachment repair.

Rhegmatogenous retinal detachment (RRD) is an acute vision-threatening pathology that requires prompt surgical intervention. Treatment options include pars plana vitrectomy with tamponade, scleral buckling, or encircling bands, combined with laser and/or cryotherapy. In the recent years, reattachment surgery has reached success rates of more than 90%.^1–3^ Despite successful surgical repair, visual acuity after macula-off RRD is not predictable, and visual outcomes vary widely. Unsatisfactory final visual acuity and/or metamorphopsia are major complains after reattachment surgery. Researchers have investigated various factors that could relate to final visual acuity after successful surgical repair,^4,5^ and such attempts have even become more popular with novel imaging devices.^6,7^ In contrast to preoperative clinical factors (such as macular status, visual acuity, or duration and area of detachment),^8,9^ postoperative findings seem to gain importance when trying to better predict the final visual outcome.^6^ Recently, visual recovery has been associated with different macular findings such as the integrity of the outer retinal microstructure,^6,10,11^ mean capillary density, and fractal dimension measured with optical coherence tomography (OCT) angiography,^12^ or a demarcation line in fundus autofluorescence (FAF) images.^13^ The retinal inflammatory response involving microglia has been linked to final visual acuity in a mouse model. Beyond lipofuscin that generates a strong signal in FAF images, in vivo quantification of molecular structures of the retina is still a challenge, and studies are mostly limited to animal experiments.

Fluorescence lifetime imaging ophthalmoscopy (FLIO) is a technique in retinal imaging that uses a picosecond pulsed blue laser light to excite endogenous retinal fluorophores and detects the subsequently emitted autofluorescence lifetime signals. A wide range of fluorophores emits signals at different wavelengths, and provides insights into the retinal molecular microstructure.^14^ Various studies have shown that FLIO is able to gather new compelling information on retinal diseases beyond the well-established imaging modalities such as OCT or FAF intensity imaging.^15^

With this study, we aimed to characterize and quantify fluorescence lifetime patterns in eyes after surgical repair after macula-off RRD.

## Methods

### Patient Selection and Examination Procedure

This observational cross-sectional study was conducted at the University Hospital in Bern, Switzerland. Local ethics committee approval was obtained, and the study was performed in accordance with International Conference on Harmonization Good Clinical Practice (ICH-GCP) guidelines, in accordance with the declaration of Helsinki. This study is registered at ClinicalTrials.gov (NCT0981148). All patients were recruited at the ophthalmology outpatient department of the University Hospital Bern, Switzerland. Written informed consent was obtained from all patients before study entry.

Patients presenting with acute macula-off RRD who underwent surgical RRD repair were identified and included in the study. Exclusion criteria were opacities of the clear ocular media that either interfered with visual acuity or image acquisition, uncontrolled glaucoma, intermediate or late stage age-related macular degeneration in the study eye, previous vitreoretinal surgery in the study eye, concomitant diseases that either interfered with visual acuity and/or fluorescence lifetimes, or any other condition that made study procedures impossible. All patients underwent pars plana vitrectomy and sulphur hexafluoride (SF_6_) gas endotamponade. Phakic patients were additionally treated with an encircling band to relax the vitreous base. Sealing of the retinal tear/hole was achieved by laser and/or cryotherapy. The respective surgeon chose the exact surgical procedure. Patients were first imaged about 1 month postoperatively after dissipation of the gas endotamponade. In selected cases preoperative images were obtained. All patients underwent a full clinical examination with measurement of the best-corrected visual acuity (BCVA; Early Treatment Diabetic Retinopathy Study [ETDRS] letters) and indirect fundoscopy. Before imaging, maximal pupil dilatation was achieved. Tropicamide 0.5% and phenylephrine HCl 2.5% were used. Spectral domain-OCT, and infrared reflectance images (Heidelberg Spectralis HRA + OCT; Heidelberg Engineering, Heidelberg, Germany), FAF and FLIO measurements were obtained. Macular pigment optical density (MPOD) was measured in a subgroup of patients (n = 24) using dual wavelength autofluorescence imaging.^16^ Macular pigment optical density measurements were correlated with fluorescence lifetime data.

### Fluorescence Lifetime Imaging Ophthalmoscope

**Fig. 1.**
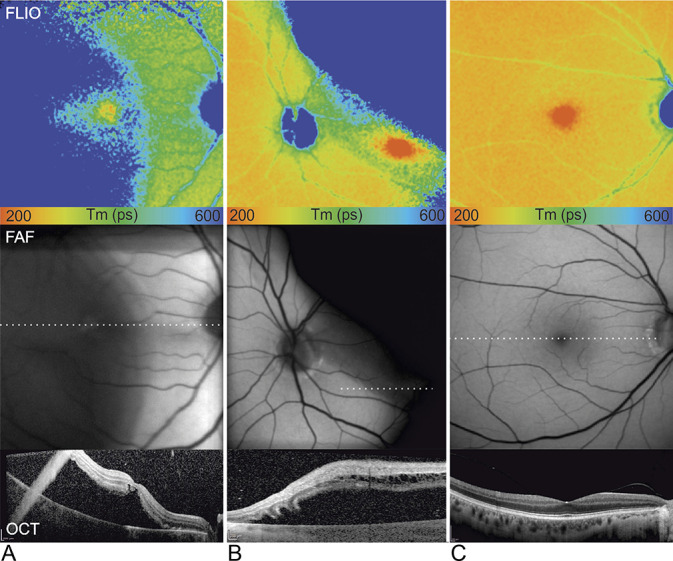
Two representative cases of macula-off RRD before surgical intervention (**A** and **B**), and unaffected FE (**C**). Color-coded fluorescence lifetime (FLIO) images of the SSC are shown with a color range from 200 to 600 ps (top panel). Fundus autofluorescence (middle panel) and OCT (indicated dotted line in FAF, bottom panel) images are displayed below.

We used an HRA Spectralis system-based fluorescence lifetime imaging ophthalmoscope to record fluorescence lifetime data (Heidelberg Engineering). The details of FLIO have been described elsewhere.^15,17^ Briefly, the principle of fluorescence lifetime data acquisition is based on the excitation of retinal autofluorescence using a 470-nm pulsed laser at 80-MHz repetition rate. The light absorbing retinal molecules are lifted to a higher energy level, and then emit photons when releasing the gained energy as they drop to their original level. The emitted photons are detected by two highly sensitive hybrid photon-counting detectors (HPM-100-40; Becker & Hickl, Berlin, Germany), and registered with time-correlated single-photon counting modules (TCSPC-150; Becker & Hickl). This process was performed in two distinct wavelength channels: a short spectral channel (SSC, 498–560 nm) and a long spectral channel (LSC, 560–720 nm). At least 1,000 photons per pixel were obtained for both channels to ensure good image quality. Examples of FLIO images are shown in Figure 1. With a measurement duration of approximately 120 seconds, an eye movement tracking system with a high-contrast confocal infrared image was used to ensure accurate tracking of detected photons within the 256 × 256 pixels frame.

### Fluorescence Lifetime Data Analysis

**Fig. 2.**
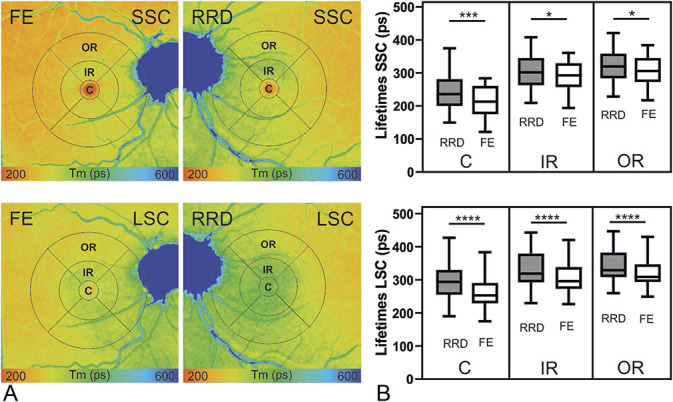
Comparison between eyes with macula-off RRD and their healthy FEs after surgical repair. **A.** FLIO images of the short (SSC) and the long (LSC) spectral channels are displayed with a color range of 200 to 600 ps and with an implemented ETDRS grid with its subregions: center (**C**), inner ring (IR), and OR. **B.** Box plot of the amplitude-weighted mean fluorescence lifetimes (Tm, ps). Analysis of variance, Tukey's multiple comparisons show significant differences between the RRD and the FE (n = 30, *P < 0.05, ***P < 0.0005, ****P < 0.0001). SSC: C; 27.8 ± 5.5 ps, *P* = 0.0003, IR; 14.8 ± 4.4 ps, *P* = 0.03, OR; 15.7 ± 4.4 ps, *P* = 0.01. LSC: C; 32.4 ± 3.7 ps, *P* < 0.0001, IR; 22.7 ± 2.7 ps, *P* < 0.0001, OR; 20.1 ± 2.7 ps, *P* < 0.0001.

Lifetime raw data was converted into exponential decay curves using SPCImage software version 7.3 (Becker & Hickl) and the chi-square test quantified the “goodness of fit.” Amplitude-weighted mean fluorescence lifetimes (Tm) were calculated using the short and the long lifetime components (T1 and T2) and their respective amplitudes α1 and α2 as described previously.^15^ The fluorescence lifetimes were further analyzed using the customized “FLIO reader” software (Artorg Center for Biomedical Engineering Research, University of Bern, Bern, Switzerland), and quantified using a standardized ETDRS grid (central subfield [C]; 1 mm, inner ring [IR]; 3 mm, outer ring [OR]; 6 mm) as shown in Figure 2.

### Statistical Data Analysis

Fluorescence lifetimes were analyzed for the SSC and the LSC separately. Data was obtained and analyzed as mean ± SEM. To compare the different regions of the ETDRS grid between the RRD eye and the patient's healthy fellow eye (FE), we performed an analysis of variance Tukey's multiple comparison test. Spearman correlation was used to relate lifetime measurements with BCVA values.

## Results

Sixty-five patients with macula-off RRD undergoing surgical intervention were identified. We excluded five eyes because of significant cataract, one eye where only preoperative images were obtained, and one eye because of insufficient image quality. The remaining 58 patients were included in this study, of these 11 were women (19%), the mean age ± SEM was 65 ± 1.64 years (range, 34–86), 41 (71%) were pseudophakic (19 with a healthy FE), and 30 (52%) patients had a healthy FE with the same lens status.

### Fluorescence Lifetime Pattern in Rhegmatogenous Retinal Detachment Before Surgical Intervention

Fluorescence lifetimes were extensively prolonged in areas where the retina was detached from the retinal pigment epithelium (RPE), as shown in Figure 1. In contrast to other “en-face” imaging modalities such as FAF and infrared images, the short fluorescence lifetimes, representing macular pigment,^17,18^ allowed to distinguish the fovea even in a detached state.

### Fluorescence Lifetimes After Surgical Intervention

For the quantitative analysis, image acquisition and clinical examination were performed at median 1.5 months after surgery (range, 0.5–50 months). The mean logMAR was 0.36 ± 0.04 (range, 0.0–1.1), which amounts to approximately 20/50 Snellen equivalent units. The Tm were assessed in the ETDRS grid subregions and are displayed in Table 1.

**Table 1.** Quantitative Analysis of Fluorescence Lifetimes

FLIO Channel	ETDRS Grid Subfield	τ_m_ (ps), All Patients, n = 58		τ_m_ (ps), RRD–FE, n = 30		τ_m_ (ps), RRD–FE, PPK, n = 19	
		Mean ± SEM	Range	Mean Difference ± SEM	*P*	Mean Difference ± SEM	*P*
SSC	C	232 ± 8	150–405	27.8 ± 5.5	0.0003	21.9 ± 4.9	0.0037
	IR	305 ± 6	206–444	14.8 ± 4.4	0.0252	10.0 ± 3.5	0.0874
	OR	322 ± 6	222–459	15.7 ± 4.4	0.0136	11.4 ± 3.3	0.0299
LSC	C	294 ± 7	190–472	32.4 ± 3.7	<0.0001	27.5 ± 4.1	<0.0001
	IR	337 ± 8	230–481	22.7 ± 2.7	<0.0001	20.6 ± 2.8	<0.0001
	OR	346 ± 8	260–458	20.1 ± 2.7	<0.0001	18.4 ± 2.9	<0.0001

C, central subfield; FE, healthy fellow eye; IR, inner ring; PPK, pseudophakic.

Furthermore, RRD eyes showed significantly prolonged fluorescence lifetimes compared with their healthy FEs, as illustrated in Table 1 and in Figure 2. The biggest difference was observed in the ETDRS grid's central subfield in both, SSC and LSC.

**Fig. 3.**
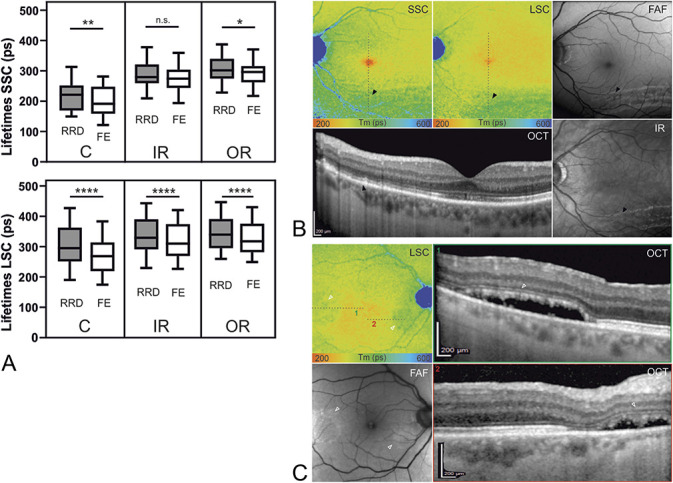
**A.** Subgroup of pseudophakic patients. Multiple comparisons show similar results as in Figure 2. (n = 19, *P < 0.05, **P < 0.005, ****P < 0.0001). SSC: C; 21.9 ± 4.9 ps, *P* = 0.004, IR; 10.0 ± 3.5 ps, *P* = 0.09, OR; 11.4 ± 3.3 ps, *P* = 0.03, LSC: C; 27.5 ± 4.1 ps, *P* < 0.0001, IR; 20.6 ± 2.8 ps, *P* < 0.0001, OR; 18.4 ± 2.9 ps, *P* < 0.0001. **B.** Example of an inferior RRD in different imaging modalities. The black arrowheads indicate the region, where photoreceptor irregularities can be observed in the OCT scan. Fundus autofluorescence and infrared (IR) images show a wave-shaped pattern in the former detached area. Corresponding regions of both, short and long spectral channel (SSC, LSC) present with prolonged FLIO lifetimes. **C.** This example shows prolonged lifetimes (hollow arrowheads in the LSC) in combination with subretinal fluid. The detached photoreceptor layer appears loosened (1) or partially destroyed (2) in the OCT.

Because fluorescence lifetime values may be influenced by the lens status,^17^ we performed a subgroup analysis on the pseudophakic patients with their healthy FEs (n = 19). These patients showed comparable results to the ones mentioned above, as shown in Table 1 and in Figure 3A. In the FLIO image in Figure 3B, the prolonged fluorescence lifetimes in the inferior retina correlated to disturbances in the photoreceptor layer in the OCT.

### Correlation With Visual Acuity

**Fig. 4.**
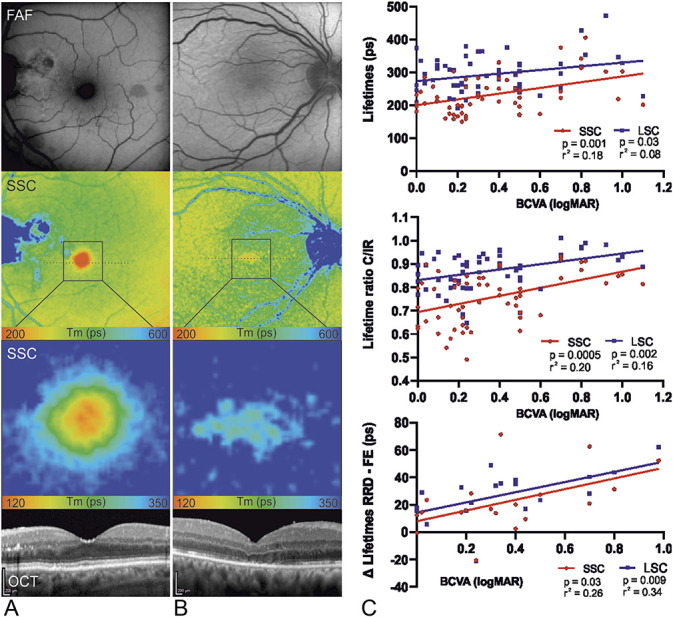
Macula-off RRD after surgical intervention. **A** and **B.** Fundus autofluorescence, fluorescence lifetime imaging (FLIO, 30 degree and enlarged area), and OCT images of the SSC of a left and a right eye are shown. **A.** The lifetime distribution appears normal, with a steep gradient between outer retina and fovea. For the enlarged FLIO image, the color range was adjusted to 120 to 350 ps to better visualize the gradient. OCT reveals a normal foveal depression with intact microstructure of the photoreceptor layer. **B.** The foveal lifetime gradient between the peripheral retina and the fovea is low. OCT shows a subfoveal disturbance in the photoreceptor layer. **C.** Correlations between quantitative analysis of the central subfield lifetimes and the BCVA (logMAR) are shown. On the top (n = 58), lifetimes (ps) of the RRD were compared with the logMAR. In the middle (n = 58), the ratio of the lifetimes of the center subfield and the inner ring of the ETDRS grid was compared with logMAR in the RRD eye. No FE data were included. On the bottom (n = 19), the difference between the FLIO lifetimes (ps) of the RRD and the healthy FE of the pseudophakic patients were compared with the logMAR. All three comparisons showed significant correlations as displayed in the figure.

Because visual acuity is the main outcome measure after RRD repair, we investigated different correlations with fluorescence lifetime data and visual acuity. Figure 4A shows an example of a patient, where good visual acuity was achieved (logMAR: 0.1, Snellen equivalent: 20/25). The fovea appeared as an area of short lifetimes, with a steep lifetime gradient between fovea and peripheral retina. In Figure 4B, the visual acuity was poor (logMAR: 0.8, Snellen equivalent: 20/125), and the gradient between fovea and peripheral retina was low. To quantify this observation, we used the ratio between the central subfield and the inner ring of the ETDRS grid: (C/IR). This ratio was used to obtain more robust information about the gradient between the fovea and the outer retina.

Fluorescence lifetimes showed significant correlations with visual acuity, as shown in Figure 4C. Short fluorescence lifetimes correlated weakly, but significantly with better visual acuity. Best correlations were observed in the SSC when looking at the absolute lifetimes and the lifetime ratio. When looking at the difference between RRD and FE, the SSC and LSC showed similar findings in pseudophakic patients.

### Fluorescence Lifetime Patterns in Relationship With Specific Optical Coherence Tomography Findings

**Fig. 5.**
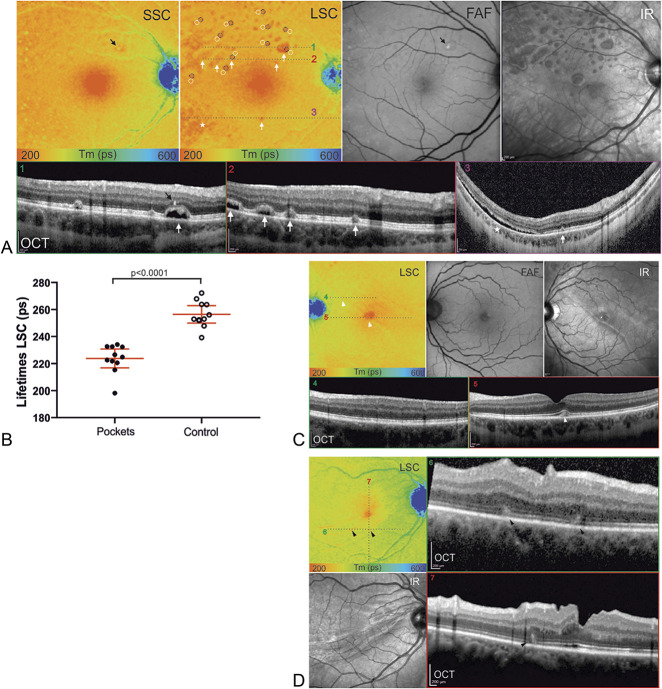
FLIO patterns after rhegmatogenous retinal detachment (RRD). **A.** Color coded (range: 200–600 ps) fluorescence lifetime (FLIO) images of the short spectral channel (SSC) and the long spectral channel (LSC), FAF intensity images, infrared images, and OCT images are shown. In the LSC, red spots of short lifetimes (white arrows) stand out. Corresponding subretinal fluid pockets can be identified in the OCT scans (1–3). A temporal inferior region of diffuse shorter lifetimes (asterisk) corresponds to diffuse subretinal fluid in the OCT scan (3) as well. One spot of prolonged lifetimes in the SSC (black arrow) corresponds to a hyperreflective focus in the OCT (1) and can also be observed in the FAF image. **B.** The FLIO lifetimes were significantly shorter in the red dots, when compared with the adjacent retina of same eccentricity, (n = 11, mean difference ± SEM: 32.65 ± 4.79 ps). **C.** In this superior temporal RRD, the LSC reveals a discrete demarcation line (white arrowheads), that corresponds to the demarcation line seen in the infrared image. Subfoveal disturbance (5, white arrowhead) can be observed, but parafoveal OCT scans (4) present normal retinal structures in the region of the demarcation line. **D.** This case shows retinal folds in the OCT scans (6/7, black arrowheads), that correspond to wave-shaped signs of short fluorescence lifetimes in the LSC. The infrared image also shows these wave-shaped signs.

In a few cases, FLIO images displayed particular patterns beyond the above-mentioned quantifiable alterations in the fovea, as shown in Figure 5. In contrast to the prolonged fluorescence lifetimes in the fovea, we found shorter lifetimes within specific areas. Four patients (7%) showed FLIO dots of shorter lifetimes that correlated to pockets of subretinal fluid, as shown in Figure 5A. However, in five cases (9%), no dots were detected despite presence of pockets on OCT. The dots exhibited shortened lifetimes compared with the adjacent retina of same eccentricity. We quantitatively analyzed the lifetimes of these dots in one representative case, as displayed in Figure 5 (mean difference ± SEM: 32.65 ± 4.79 ps, *P* < 0.0001). In addition, FLIO showed wave-shaped patterns in some patients, which correlated to retinal folds. However, one case displayed prolonged lifetimes in combination with subretinal fluid (Figure 3C), where OCT correspondingly showed an irregular and partially absent photoreceptor layer.

### Fluorescence Lifetime Imaging Ophthalmoscopy and Macular Pigment Optical Density

Macular pigment optical density at 5° eccentricity inversely correlated with fluorescence lifetimes, measured in the SSC (n = 24, *P* = 0.01, r^2^ = 0.25). No significant correlation was observed in the LSC.

## Discussion

Fluorescence lifetime imaging ophthalmoscopy is an emerging imaging technique and represents a new arrow in the quiver for earlier detection of metabolic retinal changes. Recent studies on various retinal and systemic diseases have foreshadowed the potential of FLIO.^19–23^ In past years, modern imaging and diagnostic methods have increasingly been used in research on RRD, enabling a better understanding of the postoperative macular recovery processes at the microstructural level.^6,12,24,25^ Illuminating the retina from a new perspective could therefore lead to new insights about the mechanisms of these recovery processes after macula-off RRD repair.

In our study, we show, that previously detached retina displays significantly prolonged fluorescence lifetimes and that these changes are predominantly found in the foveal area. These findings are consistent with previous studies showing prolonged lifetimes in conditions where outer retinal structures are in a pathologic state or cellular/molecular structure is disturbed.^20,21,26^ The inverse correlation of fluorescence lifetimes in the central area of the EDTRS grid with MPOD, suggest a variable amount of macular pigment loss after macula-off RRD.^18,27^

However, we also detected more subtle, but significantly prolonged lifetimes in previously detached retina in the inner and OR of the ETDRS grid. These findings suggest that the differences in lifetimes may not be solely explained by disturbances in macular pigment. Considering the significant correlation between foveal lifetimes and visual acuity in the RRD eyes, other factors that stand in a closer relation to visual acuity than macular pigment may additionally play a role.^28^ For example, Park et al showed that the microstructure of the photoreceptor layer stands in close relation to the visual acuity recovery. The presence of a “foveal bulge” and the length of the photoreceptor inner and outer segments were associated with visual acuity recovery.^6^

A possible explanation for prolonged lifetimes peripheral to the fovea in previously detached retina after successful RRD repair may be, that morphologic retinal microstructure alterations and impairments (either caused by the detachment itself, or the following surgery) cause rarefication of photoreceptors, thus decreased retinal byproducts, which in turn lead to prolonged fluorescence lifetimes. However, as the exact source of the FLIO signal remains obscure, other factors, such as changes in the RPE or structural alterations of other layers, may be responsible for these findings.

Another finding that merits further discussion is the markedly prolonged fluorescence lifetime found preoperatively in the area of detached retina (Figure 1). We can only speculate about the provenience of the long fluorescence lifetimes in the area of detached retina. One possible explanation may be that metabolic changes within the detached retina cause this prolongation. Apart from microstructural and/or metabolic changes occurring in the detached retina, another possible explanation for the prolonged lifetimes may be due to a shift of the ratio of fluorophores coming from the retina and RPE respectively. Because the retina in the detached state is not imaged perpendicularly, the retina may contribute over proportionally to the FLIO signal. Another explanation could be, that because of the massive subretinal fluid and consecutive increase of scatter or increased absorption by the detached retina, the excitation laser does not reach the RPE properly and less signal from the RPE is detected. Furthermore as seen in Figure 1 as well, the border zone of the prolonged lifetimes in FLIO is not completely congruent with the findings on OCT, suggesting that the lifetimes are still within the “normal” range in the border zone of already detached areas. Once again, this may be due to absence of microstructural/metabolic changes in this border zone or due to the absence of optical artefacts in this zone.

Absolute values of the lifetime data have a broad range, even in healthy eyes,^17^ and therefore quantitative analyses are difficult and must be interpreted with caution. Comparing the RRD eyes with their healthy FEs largely corrected for this variance among different subjects. This furthermore enabled us to obtain a better correlation with postoperative BCVA (Figure 4). The intrinsic ratio “C/IR” not only allowed to correct for intereye differences, but also supported our initial results with a very good correlation with postoperative BCVA. These correlations are consistent with a previous study, where fluorescence lifetimes were measured one month after vitrectomy, in patients with macular holes and where short fluorescence lifetimes were also associated with better visual acuity.^29^ Although the disease mechanism is different, our study confirms this association for patients after RRD repair.

Apart from general fluorescence lifetime alterations, we detected shorter lifetime patterns in areas of subretinal fluid pockets and retinal folds. Dysli et al^21^ described short fluorescence lifetimes in areas with subretinal fluid in central serous chorioretinopathy. These findings were interpreted as accumulation of photoreceptor segments in the detached areas. In this study, we suspect a similar mechanism that may lead to the shorter fluorescence lifetimes observed in our patients. Because there were several subretinal fluid pockets where no FLIO signal alteration was detected, we assume that the FLIO signal may only appear in the later evolutional stages of these pockets, similar to what has been described in central serous chorioretinopathy. However, short fluorescence lifetimes may also derive from other factors such as blood or serum components.^30^

In spite of the many insights that FLIO has already made possible in the recent years, investigators face several limitations when using it. A broad range of fluorophores exists within the retina, and the source of the FLIO signal is not yet conclusively understood nor identified. Data interpretation strongly depends on software improvement, and the complex structure of the data including its multiexponential decay requires significant expertise, which makes use in daily routine difficult at this time. The “en-face” nature of the signal implies several problems as well. In addition to the BCVA correlation shown in our study, this data would benefit from additional parameters such as microperimetry or identification of photoreceptor density with adaptive optics that would further support our findings. Additional and more study-specific limitations include the lack of longitudinal data and correlation with visual acuity recovery.

Nevertheless, FLIO represents an interesting novel diagnostic tool in the investigation of macula-off RRD. Our findings confirm that molecular structure of the fovea is altered and stands in relationship with visual outcome after surgical intervention in RRD patients. We detected certain parallels to previously described findings from other retinal diseases, which suggest similarities on a molecular basis. Longitudinal studies are important to further investigate possible predictive factors for long-term functional outcome in these patients.
